# Switch or stay? Automatic classification of internal mental states in bistable perception

**DOI:** 10.1007/s11571-019-09548-7

**Published:** 2019-07-19

**Authors:** Susmita Sen, Syed Naser Daimi, Katsumi Watanabe, Kohske Takahashi, Joydeep Bhattacharya, Goutam Saha

**Affiliations:** 1 Department of Electronics and Electrical Communication Engineering, Indian Institute of Technology Kharagpur, Kharagpur, 721 302 India; 2 Department of Intermediate Art and Science, Waseda University, Tokyo, Japan; 3 School of Psychology, Chukyo University, Nagoya, Japan; 4 Department of Psychology, Goldsmiths, University of London, London, UK

**Keywords:** Internal mental states, Bistable perception, MEG, Single-trial classification, Source reconstruction, SVM, ANN

## Abstract

The human brain goes through numerous cognitive states, most of these being hidden or implicit while performing a task, and understanding them is of great practical importance. However, identifying internal mental states is quite challenging as these states are difficult to label, usually short-lived, and generally, overlap with other tasks. One such problem pertains to bistable perception, which we consider to consist of two internal mental states, namely, transition and maintenance. The transition state is short-lived and represents a change in perception while the maintenance state is comparatively longer and represents a stable perception. In this study, we proposed a novel approach for characterizing the duration of transition and maintenance states and classified them from the neuromagnetic brain responses. Participants were presented with various types of ambiguous visual stimuli on which they indicated the moments of perceptual switches, while their magnetoencephalogram (MEG) data were recorded. We extracted different spatio-temporal features based on wavelet transform, and classified transition and maintenance states on a trial-by-trial basis. We obtained a classification accuracy of 79.58% and 78.40% using SVM and ANN classifiers, respectively. Next, we investigated the temporal fluctuations of these internal mental representations as captured by our classifier model and found that the accuracy showed a decreasing trend as the maintenance state was moved towards the next transition state. Further, to identify the neural sources corresponding to these internal mental states, we performed source analysis on MEG signals. We observed the involvement of sources from the parietal lobe, occipital lobe, and cerebellum in distinguishing transition and maintenance states. Cross-conditional classification analysis established generalization potential of wavelet features. Altogether, this study presents an automatic classification of endogenous mental states involved in bistable perception by establishing brain-behavior relationships at the single-trial level.

## Introduction

The human brain has a unique ability to perform various cognitive processes that can be represented by different and potentially distinct cognitive states. In the last decade, there has been intense interest in exploring the possibility to decode underlying cognitive states from the observed brain signals measured by neuroimaging techniques (Haynes and Rees 2006; Richmond et al. 2012). For example, studies performed decoding of mental states underlying resting state, recalling events, performing mathematical computation and singing (Shirer et al. 2012), decoding of speech or video quality perception (Porbadnigk et al. 2013; Acqualagna et al. 2015), detecting the level of alertness (Hsu and Jung 2017) from their ongoing brain activity. Understanding and identifying the mental states through brain responses can be of great importance in human-machine interaction and brain-computer interfaces (BCI) applications (Calvo et al. 2014). Most studies deal with decoding the perception of objects and visual images, namely decoding the perceptual states while visualizing face or any objects (Allison et al. 1994; Kanwisher et al. 1997), house or visual scenes (Epstein and Kanwisher 1998), as well as orientation, location, color, and direction of motion of objects (Carlson et al. 2011; Haynes and Rees 2005; Kamitani and Tong 2005). These processes are dependent upon the information contained in the visual stimuli.

**Fig. 1:**
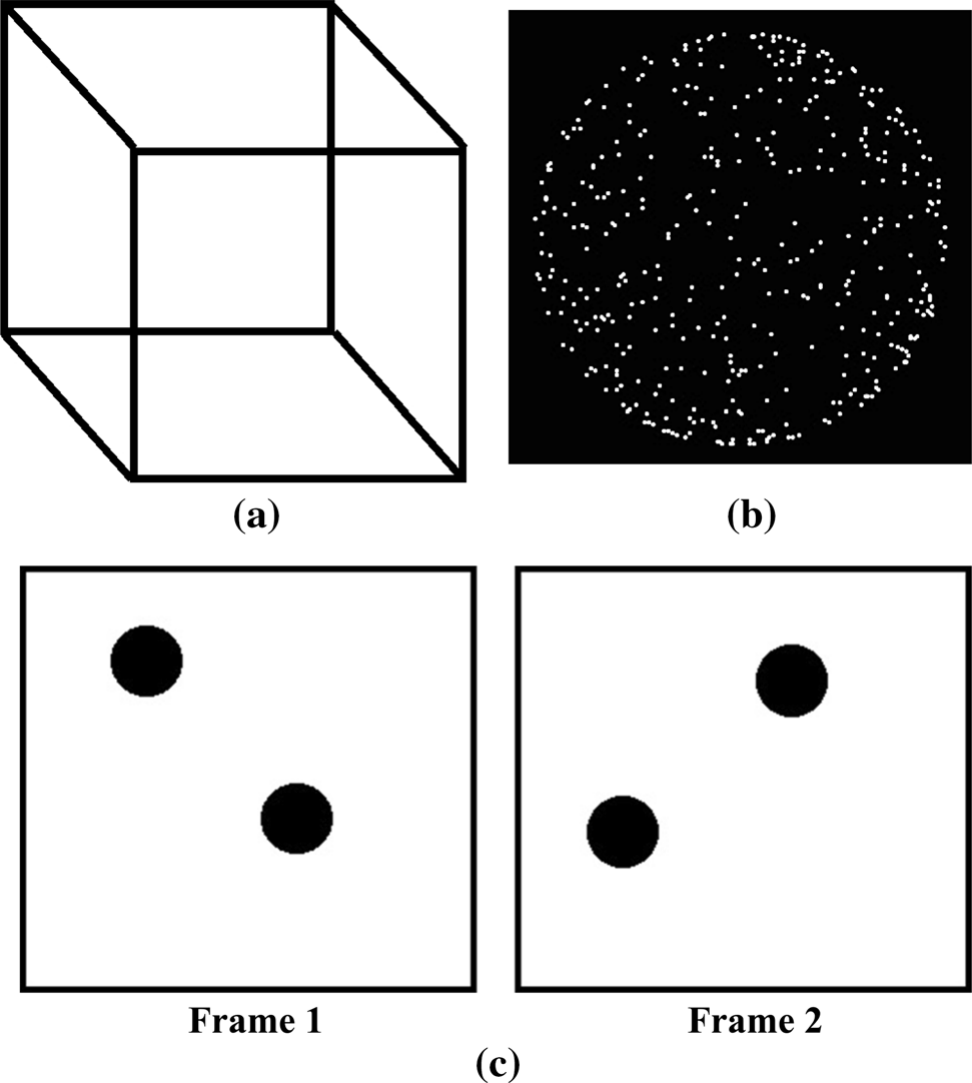
Bistable visual stimuli used in this experiment. **a** Necker Cube (NC): front surface flips with time; **b** structure-from-motion (SFM): sphere made of dots seems to move in anticlockwise or clockwise direction; **c** stroboscopic alternative motion (SAM): frame1 and frame2 are presented in such a manner that there lies an ambiguity in the direction in which dots move—horizontally or vertically

Further, decoding of spontaneously changing dynamical states, that come close to the practical scenario is also studied (Haynes and Rees 2006). Such stimuli include ambiguous visual stimuli, e.g., binocular rivalry or bistable figures (Fig. 1), which can be perceived with two interpretations but without any concomitant change in the external sensory input. Hence, there is a sharp dissociation between consistent visual stimuli and fluctuating conscious awareness (Blake and Logothetis 2002). In this study, we considered the problem of decoding the internal mental states involved with bistable perception.

The studies that use bistable stimuli, mostly analyze the brain responses of the alternating states of the perception (Knapen et al. 2011; Sterzer et al. 2009; Isoglu-Alkaç et al. 2000). Among these studies, a large number uses fMRI data (Knapen et al. 2011; Sterzer et al. 2009), but there are also a few studies using EEG/MEG. For example, Isoglu-Alkaç et al. (2000) have experimented with Necker cube as visual stimuli and have compared the alpha band (8–16 Hz) activity in two-time windows: 800–440 ms and 440–80 ms before the button press at perceptual change. They have found a noticeable decrease in alpha activity from former time window to the latter one. Interestingly, another study (İşoğlu-Alkaç and Strüber 2006) reported a decrease in only the lower alpha band (6–10 Hz) power, while the upper alpha band (10–12 Hz) activity remained unchanged. The MEG alpha band activities during the perceptual reversal in case of exogenously and endogenously induced reversal were also compared (Strüber and Herrmann 2002). Endogenous reversal of perceived motion direction takes place spontaneously in the presence of constant ambiguous stimuli, whereas, exogenous reversals are driven by a change in external stimuli. In both cases, participants were instructed to press a button whenever a change in the perceived motion direction occurred. The authors have reported that in the case of an exogenously induced reversal, alpha activity (10 Hz) started decreasing between 300 and 200 ms before the button press. On the contrary, in the case of an endogenously induced reversal, alpha activity decreased within 1000 ms before the button press. In another study (Başar-Eroglu et al. 1996), it is observed that high-frequency gamma band (30–50 Hz) oscillations were dominant in the right frontal cortex within 1000 ms before the button press. Recently, Kloosterman et al. (2015) studied motion-induced blindness, another perceptual illusion under identical sensory input, and reported that this illusion was associated with the beta band (12–30 Hz) oscillations over visual cortex out of top-down modulation.

These studies suggest that the large scale brain oscillations and their temporal dynamics are associated with perceptual switching bistable perception. This further suggests that the underlying cognitive states are dynamic. In bistable perception, the subjective perception alternates between two interpretations spontaneously and without any change in the visual input; besides two perceptual states, the processing itself contains separate internal mental states—transition and maintenance (Rees 2001). During the state of transition, perception switches from one perception to another. On the other hand, one perception remains unaltered throughout the maintenance state. The earlier studies on bistable perception, albeit informative and explanatory towards revealing the neuronal mechanisms underlying ambiguous visual perception, has so far not aimed to classify internal mental states, transition and maintenance, on a single-trial basis. The task of distinguishing these states through brain signals is not trivial as the underlying processes for bistable perception overlap with those of simple perception (Long and Toppino 2004), the rapid occurrence of transition further makes it difficult to analyze on a single-trial basis. The principal aim of this study was to classify the internal mental states of the brain that goes through the states of transition and maintenance around the moment of the perceptual switch during bistable visual perception on a single-trial basis.

The neuromagnetic brain responses (MEG) were recorded from eleven healthy participants while they were presented with ambiguous bistable visual stimuli. We used a machine-learning framework involving feature extraction, dimensionality reduction, and classification. The complex Morlet wavelet transform was used to extract spectral features that capture the spatiotemporal dynamics of large-scale brain oscillations. Three types of features were proposed that capture the spatiotemporal brain activity at different spatial scales—overall (global), hemispheric, and regional (local) brain activity, respectively. For dimension reduction, we followed the Principal Component Analysis (PCA) based approach. Finally, we validated our classification performance using two widely used classifiers, namely Support Vector Machine (SVM) and Artificial Neural Network (ANN).

This study addresses several challenges associated with decoding the internal mental states involved in bistable perception. Our contributions are as follows.

1. The internal mental states changes spontaneously, that, are not locked to a trigger in external stimuli. This makes labelling them difficult. Here, we considered the MEG signal before and after the button press to define the transition and maintenance state. The reason is that the participants were instructed to report the perceptual switch while they were viewing bistable stimuli. It is likely that the change in perception had occurred a little ahead of the time the perceptual switches were reported.
2. The transition state is short-lived as compared to maintenance state. It is of interest to determine the shortest duration of the transition state, that distinguishes it from the maintenance state. For this purpose, we adopted the machine learning framework to infer an effective duration of the transition state.
3. We investigated if the underlying states, as captured by the decoding performance, differed across the type of bistable stimuli considered. For this, we used three types of bistable stimuli (Fig. 1): Necker cube (NC), apparent motion or stroboscopic alternative motion (SAM) and structure-from-motion (SFM).
4. We propose a framework to characterize the variation of the maintenance state with time. As mentioned earlier, the maintenance state extends over a longer duration than the transition state. Here, we investigated if the underlying neural representation remains stable over the maintenance period; the proposed framework captures the intrinsic temporal fluctuations of maintenance state representation, in terms of its decoding performance.
5. We localized the underlying brain sources whose activity is modulated by the internal mental state under consideration.
6. We investigated the neural abstraction in discriminating transition and maintenance states across different stimulus types, that is, whether the brain responses captured by wavelet features are generalized across different stimulus types. For this, we utilized cross-conditional classification approach where data of one stimulus type was used for training and testing it with the data from other stimulus types.

Altogether, this work designed and presented a direction to characterize and decode internal mental states involved in bistable perception at the single-trial level.

## Materials and methods

### Dataset

#### Participants

In this experiment, MEG data were recorded from eleven adult participants. All participants were right-handed, neurologically healthy and had normal vision. Written informed consent was obtained from all participants. The experimental protocol was approved by the Local Ethics Committee and followed the declaration of Helsinki.

#### Stimuli

Three types of bistable stimuli were used, namely: Necker cube (NC), apparent motion or stroboscopic alternative motion (SAM) and structure-from-motion (SFM) (Fig. 1). The perceptual interpretation of Necker cube oscillates between two different recessional surfaces. In the case of SAM, there is an ambiguity about the direction in which the dots move—horizontally or vertically. The SFM used here was a sphere comprised of dots that seems to be moving. While observing SFM, the perceived direction of rotation appears to flip over time.

#### Procedure

Visual stimuli were projected onto a rear screen, located 32 cm from the participant’s eyes by a projector (PG-B10S; SHARP, Osaka, Japan) via a mirror. The visual stimuli were generated by using C++ and OpenGL. The size and luminance were far above visual threshold. The rotating sphere consisted of 200 dots. The basic experimental setting was identical with a previously published article (Kondo et al. 2010). There were 18 trials (3 [stimulus type] × 2 [stimuli presentation type] × 3 repetition) for each participants. The duration of each trial was 60 s. In this duration, bistable figures were shown in two formats: (i) in a continuous format, the stimulus was presented continuously over this 60 s duration, and (ii) in discrete or blanking format, the stimulus was presented for alternative 3 s with and was off for 3 s. During the alternative 3 s when stimuli were not presented, only the blank screen was there. The blanking was introduced to modulate the rate of perceptual switch (Leopold et al. 2002). Participants pressed a button whenever they experienced a flip in their perception, from one perceptual state to the other, e.g. in SFM, a switching from clockwise perception to anticlockwise perception or reverse. Since there were three types of stimuli and each was presented in two formats, there were six conditions in total, and each condition was repeated three times. Thus, eighteen such sequential data blocks of 60 s were acquired from each participant, and the block of stimuli was randomized across all participants for experimentation. Subject wise trial statistics across different stimuli are presented in Table 1. It is apparent that the variability across participants was correlated across stimulus.

**Table 1:** Number of perceptual switch for different condition for each subjects

	NCC	SAMC	SFMC	NCB	SAMB	SFMC
Subject 01	23	4	34	27	2	29
Subject 02	21	0	3	18	6	3
Subject 03	40	28	17	31	0	3
Subject 04	50	17	13	8	10	3
Subject 05	38	3	20	24	0	2
Subject 06	50	6	15	16	1	1
Subject 07	15	0	6	11	0	1
Subject 08	28	9	20	25	17	12
Subject 09	58	0	16	24	0	3
Subject 10	20	12	20	18	11	13
Subject 11	25	6	7	26	7	10
Total	368	85	171	228	54	80

#### Data acquisition and preprocessing

MEG data were recorded with a 224-channel superconducting quantum inference device (SQUID) whole-head coaxial gradiometer Yokogawa MEG system in a magnetically shielded room. Out of these 224 channels, only 160 MEG sensors were used to record data. Signals were sampled at 1000 Hz. Data were subsequently band-pass filtered in the frequency range of 1-80 Hz, and a notch filter was applied to remove power line interference. The eye blink and heartbeat artifacts were removed with independent component analysis (ICA). Further, data containing saccade and muscle artifacts were rejected by visual inspection. All pre-processing were performed using FieldTrip MATLAB toolbox (Oostenveld et al. 2011).

#### Epochs formulation

**Fig. 2:**
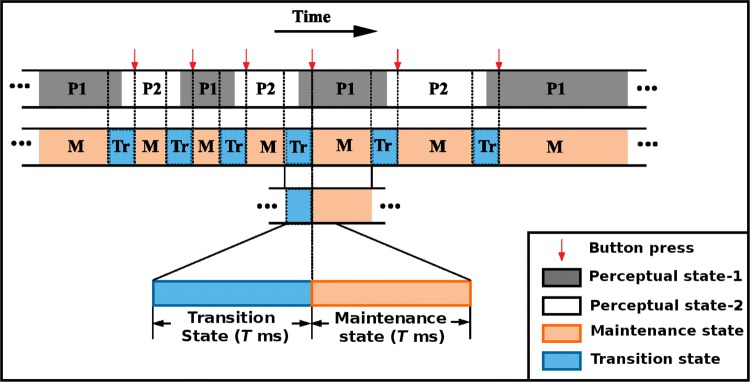
One representative time block while a bistable stimulus was presented. Perception of individual changes between two perceptual states P1 and P2. Red vertical arrows indicate the time instants at which the participant reported the perceptual switch. Reaction time, which is the time difference between the endogenous perception change and response time, varies from person to person as well as trial to trial. The transition state is considered to be of *T* ms (which is much longer than the reaction time, Kosinski 2008) prior to each button press so that it captures the switch in perception. Segments of duration *T* ms after each reported perceptual switch were considered as maintenance state, during which the perception remains stable. (Color figure online)

Two interpretations of bistable stimuli alternately dominate in time. This leads to two different perceptual states, namely, P1 and P2 (Fig. 2). The participants were instructed to report the perceptual switch while they were viewing bistable stimuli. It is likely that the change in perception had occurred a little ahead of the time the perceptual switches were reported. This is due to the reaction time, which varies from person-to-person as well as trial-to-trial. However, besides these two broad categories of perceptual states, there exist two different internal mental states—maintenance and transition (Fig. 2). One of the perceptions remains stable during the maintenance state, whereas during the transition state, the perceptual change or switching takes place. Thus, the transition state is comparatively shorter than the maintenance state. To develop the analysis framework, we considered two time periods of equal length (to have same dimension of feature vector for both the classes) around the moment of each perceptual switch: (i) transition state, a window of length *T* ms (1800 ms) before the button press, and (ii) maintenance state, a window of the same length after the button press. We varied the window length and performed shifting the window for further analysis. We also treated this as a classification problem where transition and maintenance states were treated as two different classes. The considered transition and maintenance states are shown in Fig. 2. The trials with overlapped maintenance state with the next transition or overlapped transition state with previous maintenance state were rejected from the analysis. Table 2 provides the number of available trials for six conditions. Note that, in our framework, no distinction was made between P1 and P2 states, and they were randomly placed in a nearly equal number in the pooled data having transition and maintenance labels. Thus, the classification accuracies reported in this study for transition and maintenance is irrespective of perceptual state (P1 or P2) before and after the button press.

**Table 2:** List of different stimuli and their presentation mode with number of available trials. Trials were pooled across all participants

Type of stimuli and presentation mode	Number of trials
NC presented continuously (NCC)	165
SAM presented continuously (SAMC)	56
SFM presented continuously (SFMC)	133
NC presented with blanking (NCB)	158
SAM presented with blanking (SAMB)	51
SFM presented with blanking (SFMB)	65

### Wavelet based features extraction

In this study, features were derived to capture the spectral content of the large-scale brain oscillations as they have been successfully used in the sensory and cognitive processing (Buzsáki and Watson 2012; Ward 2003) including bistable perception (İşoğlu-Alkaç et al. 1998; Isoglu-Alkaç et al. 2000; Strüber and Herrmann 2002). We used the wavelet transform that decomposes the signal into a number of scaled and shifted version of the basis function often called, ‘mother wavelet’. There exist several wavelet transforms depending on the characteristics of the mother wavelet. To extract the desired information, a mother wavelet is chosen in such a way so that it matches with the waveform of the signal. Morlet wavelet has been chosen in a number of EEG and MEG studies (Isoglu-Alkaç et al. 2000; Ghuman et al. 2011). In this work, therefore, we used complex Morlet wavelet (Cohen 2014) for feature extraction which is mathematically represented by a complex sine wave, multiplied by a Gaussian window as shown in Eq.(1).

1 $$\begin{aligned} cmw(t,f) = A e^{-t^{2}/2s^{2}} e^{^{i2 \pi ft}} \quad for \quad t = 1,2,\ldots ,T \end{aligned}$$

where $A = 1/{(s\sqrt{\pi })}^{1/2}$ is the frequency band specific scaling factor, *s* is the standard deviation of the Gaussian wave, *f* is the frequency of the wavelet basis, $t=nT_{s}$, $T_{s}$ is the sampling period and *n* is the index of samples. Here, *f* was logarithmically scaled between 1 Hz and 80 Hz and sampling frequency, $1/T_{s}$ was 1000 Hz.

Let, $X_{g}(t)$ be the $g$th time series. We define $Y_{g}(t,f)$ as the wavelet transform of signal $X_{g}(t)$ which can be expressed as the inner product between the signal $X_{g}(t)$ and complex Morlet wavelet *cmw*(*t*, *f*).

2 $$\begin{aligned} Y_{g}(t,f) = {<}X_{g}(t),cmw(t,f){>}\end{aligned}$$

In this study, the spectral information was divided into six frequency bands: delta band (1–4 Hz), theta band (4–8 Hz), alpha band (8–13 Hz), beta band (13–30 Hz), lower gamma band (30–50 Hz) and upper gamma band (50–80 Hz). To obtain frequency band specific activity, square operation was performed on $Y_{g}(t,f)$ followed by averaging over the frequency points that lie in that particular frequency band. This is defined by Eq.(3).

3 $$\begin{aligned} Z_{g}(t,b) = \frac{1}{N_{B_{b}}}\sum _{\forall f\in b^{th} band} |Y_{g}(t,f)|^{2} \end{aligned}$$

where $N_{B_{b}}$ denotes the number of frequency bins in the $b^{th}$ band ($b = 1,2,\ldots ,6$).

The averaging of the band-specific energies over time series (sensors) was performed in three ways to extract three types of features. For extracting global features, the averaging was performed over all the considered sensors. The left and right hemispheric features were extracted by averaging over the sensors that belong to the left and right hemisphere, respectively. To derive local features, sensors were grouped in 10 clusters (left and right frontal (LF, RF), left and right fronto-temporal (LFT, RFT), left parieto-temporal (LPT), right occipito-temporal (LOT), left and right parietal (LP, RP), posterior parietal (PP), left occipital (LO)) by applying Ward’s clustering algorithm (Ward 1963) to the position of the sensors. The aim of Ward’s algorithm is to construct the clusters in such a way that within-cluster variance is minimized. Thus, the local features were computed in 10 cortical regions by averaging over sensors that belonged to a particular cluster. As the perceptual moment was indicated by a button press, there is a need to exclude the motor-related activity to avoid its influence on the classification performance (Wang et al. 2013). Therefore, while computing the features, in the first place, we excluded 36 sensors from somatosensory and motor regions of both the hemispheres.

**Fig. 3:**
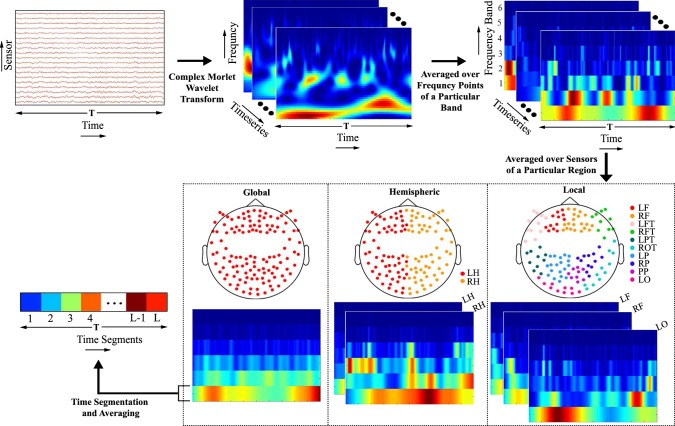
Different steps involved in the feature extraction process. Complex Morlet wavelet transform was applied on the *T* ms length time series data. To obtain band-specific energy, the wavelet coefficients of wavelet transform were squared and averaged over the frequency points that belong to a specific frequency band [Delta (1–4 Hz), Theta (4–8 Hz), Alpha (8–13 Hz), Beta (13–30), lower Gamma (30–50 Hz) and upper Gamma (50–80 Hz)]. Three types of the feature were extracted—global feature, hemispheric (left and right) feature and local feature. To extract global, hemispheric and local features, frequency band energies were averaged over all the sensors, sensors that belonged to particular hemisphere and sensors that belong to a particular region, respectively. In all these three cases, the sensors from the motor cortex were excluded. The temporal dynamics of these features were approximated by diving *T* ms into *L* segments and averaged over the time points of each segment separately

In the next step, the total time duration of *T* ms was divided into *L* number of segments, and for each segment, power was computed. The parameters *T* and *L* were empirically determined (see “Results” section). While the global and hemispheric features approximated the temporal dynamics of the power in six frequency bands over the whole cortical and hemispheric region, respectively, the local features captured the same but retaining individual attributes of 10 cortical regions. The detailed block diagram of the feature extraction technique is shown in Fig. 3.

### Source reconstruction

The underlying neural sources were reconstructed using Linear Constraint Minimum Variance (LCMV) method (Van Veen et al. 1997). In this method, a bank of spatial filters was designed. Filter weights were chosen in such a way so that, it passed brain electrical activity from a specified location while attenuating activity originating at other locations by minimizing the filter output power subjected to a linear constraint.

For source analysis, forward models were constructed based on a standard structural T1-weighted MRI template “Colin27” (Holmes et al. 1998). For beamformer solution, the covariance matrix was calculated by considering the epochs—1800 ms to 0 ms and also 0 ms to 1800 ms where 0 ms indicated the time instant when the perceptual transition was reported. The dipoles were assumed to be located at the voxels within the head boundary (the only grey matter was considered) on a 3D grid with 5 mm spacing. This resulted in 11,780 positions where sources were to be localized. Subsequently, beamformer filters were designed to pass the signal of interest at these locations and attenuate the rest. Time series data were then projected through the resulting beamformer coefficients to produce time courses. According to Automated Anatomical Labelling (AAL), the grey matter of the brain was divided into 116 regions. For each brain region, the voxels that belong to that region were identified, and the corresponding time series were grouped. We considered the principal eigenvector of the grouped time series that represented a particular region (Friston et al. 2006). Thus, there were 116 time-series, each representing one region. Twenty-seven out of 116 regions were not considered which are often involved with the following functions: finger movement; contralateral finger, hand, and wrist movement; movement initiation and movement preparation^1^. Thereby, 89 time-series were considered for further processing. The wavelet features were extracted from these 89 time-series by following the method explained in “Wavelet based features extraction” section. However, the step of averaging over time series was not considered in this case, since we wanted to capture the activity in these 89 brain regions, individually.

### Classifier

Due to the wide variability of perception switching rate across individuals, the number of trials differed from participant to participant, resulting in very few trials for some cases. Thus, personalized models were not considered, and we only performed classification at the group level by pooling trials across all participants (Table 2). We used SVM (Vapnik 2013) classifier with Radial Basis Function (RBF) as the kernel function, and also, ANN classifier. The application of SVM and ANN classifier has been found in various studies related to the classification of to neuro-signal (Alimardani et al. 2013; Subasi 2005). For ANN classifier, the number of nodes at the hidden layer was fixed at 10. Scale-conjugate gradient backpropagation algorithm was used to train the model. Mean square error was set to 10^−5^, and the hyperbolic tangent sigmoid transfer function was used as the activation function.

The performance of the classifier was evaluated using 10-fold cross-validation. It is considered more reliable compared to leave-one-out cross-validation (Varoquaux et al. 2017). Of note, the data to train the classifier and the data to test its performance were mutually exclusive. The performance of the classifier was measured by accuracy, sensitivity, and specificity; where sensitivity and specificity quantify how accurately the model was able to detect the transition, and maintenance states, respectively. All analysis was performed using customized scripts using MATLAB 2013a.

## Results

We aim to classify the two internal mental states that involve different complex cognitive processing. In a number of earlier studies, the oscillatory activities in the alpha, beta and gamma band were found to play important role in bistable perception as well as complex cognitive processing (Piantoni et al. 2017; Lange et al. 2014; Piantoni et al. 2010; Okazaki et al. 2008; Kloosterman et al. 2015; Keil et al. 1999; Başar-Eroglu et al. 1996). So, we validated this on our data by using Bonferroni corrected t-test between trials from transition and maintenance states of whole epoch for all six frequency bands considered in this study. The difference between the transition/maintenance states was found to be significant ($p < 0.05/6 = 0.0083$) for the following bands: alpha [$t(627)=-\,4.9256$, $p<0.0083$], beta [$t(627)=-\,10.3515$, $p<0.0083$], lower gamma [$t(627)=-\,12.3498$, $p<0.0083$], and upper gamma [$t(627)=-\,6.9995$, $p<0.0083$]. Thus, we used features from these fours bands to predict the mental states on a single-trial basis.

### Effective duration of transition state

**Fig. 4:**
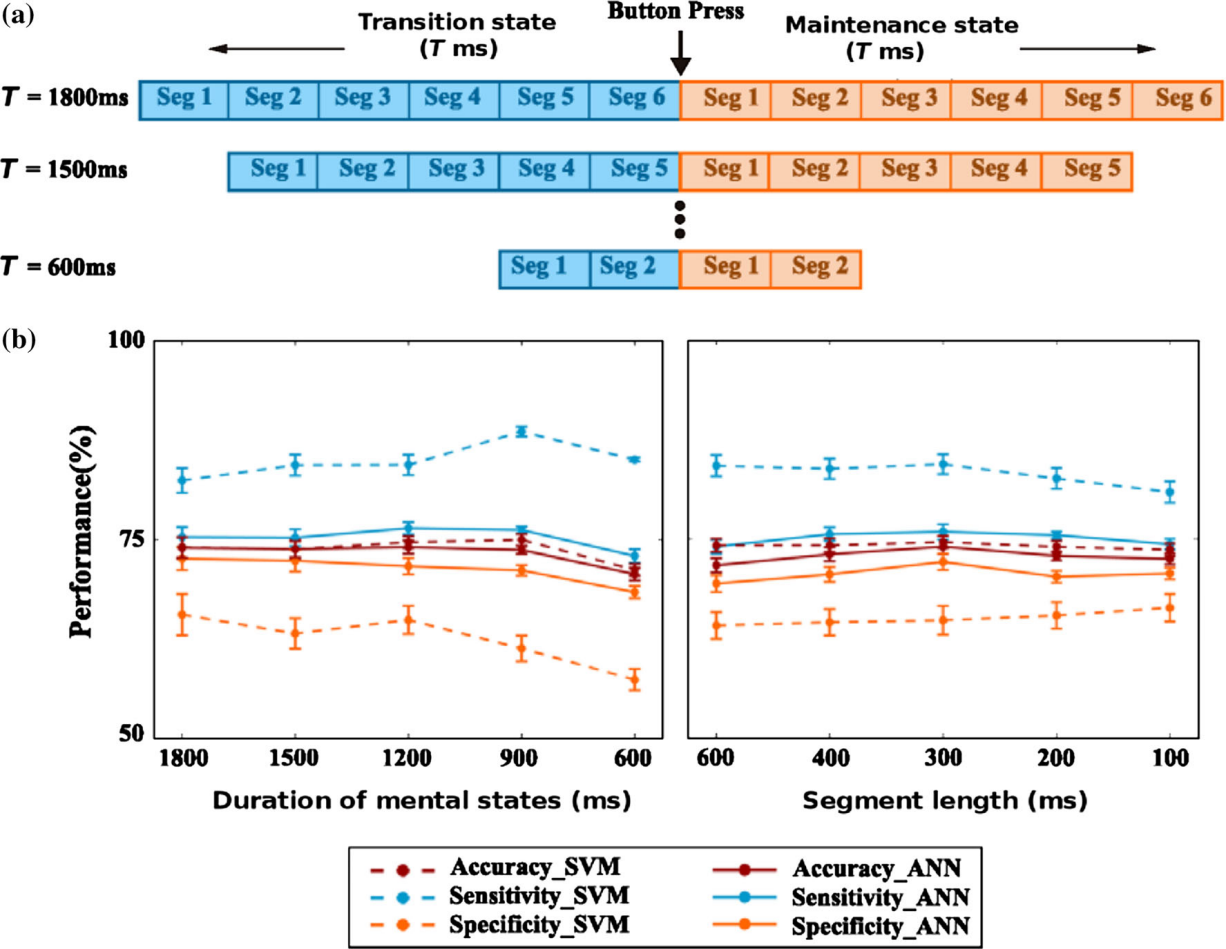
**a** The length of the transition and maintenance state was varied from 1800 to 600 ms and accordingly, classification performance of the extracted features (alpha, beta, lower gamma, and upper gamma) were evaluated for each condition, separately. The result of classification was provided in (**b**). The left panel shows the accuracy, sensitivity, and specificity varied with the different length of transition and maintenance state, using both SVM and ANN classifiers. Here, sensitivity and specificity refer to how accurately the transition and maintenance state were classified, respectively. The right panel shows the classification performance for different segment lengths. The plot represents the mean accuracy, sensitivity and specificity of the six conditions with the standard error of the mean (SEM) shown by the error bars

The participants indicated perceptual reversal by pressing a button. However, the reaction time might vary across participants as well as from trial-to-trial. Additionally, some transition occurs instantaneously whereas “other transitions comprise the dynamic mixture of both the percepts for variable periods before one percept dominates completely” (Knapen et al. 2011), thus leading to a variable duration of the transition state. To determine the effective time-duration that best represents the transition state, we considered the different extents of transition state varying from 600 to 1800 ms in steps of 300 ms. To retain the same feature dimension for both the classes, the length of the maintenance state was kept the same as the length of the transition state. Figure 4a shows the different transition and maintenance states considered for the analysis. For each case, the global feature was extracted from the alpha, beta, lower gamma, and upper gamma frequency band using segment length $l= T/L$ of 300 ms and then used in classification. Table 3 shows the feature dimension for different length of the transition state. The classification performance, i.e. accuracy, sensitivity, and specificity concerning the length of the transition state, are shown on the left panel of Fig. 4b.

**Table 3:** Variation of feature dimension with duration of mental states (length of each segment = 300 ms)

Duration of mental states (ms)	No. of segment (L)	Feature dimension (4 bands × L)
1800	6	24
1500	5	20
1200	4	26
900	3	12
600	2	8

Since accuracy, sensitivity, and specificity for the length of 1200 ms were comparatively good and adequate for all the conditions and both classifiers, it was considered as the effective length of the transition state and used for further analysis. Next, we analyzed the performance variation with respect to the segment length, which was kept 300 ms throughout the previous analysis. Here, we varied the segment length from 100 to 600 ms (in steps of 100 ms), and the extracted features were used for classification (right panel of Fig. 4b). The classification performance remained relatively stable across various segment lengths. Since the segment length of 300 ms yielded the best accuracy, it was considered for further analysis.

For classification of mental states, three types of features were considered: (1) global features, (2) hemispheric (left and right hemispheric) features, and (3) local brain region-specific features. Along with the six conditions listed in Table 2, we also pooled the trials irrespective of types of condition, and their presentation mode is referred as ‘ACT’ (all conditions together) in this paper.

### Single trial classification

We computed global, hemispheric, and local features in the alpha, beta, lower gamma, and upper gamma frequency band for transition and maintenance states of a duration of 1200 ms. The feature dimension for global, hemispheric, and local features were 16, 16, and 160, respectively. A 10-fold cross-validation was performed to evaluate the performance. In the case of local features, we used PCA to reduce the feature dimension. The PCA was applied to the training data of 9 folds, and the new dimension was determined from the principal components that capture $C_{v}\%$ (cumulative variance) of the total variance. The cumulative variance was varied from 95 to 99.9%, and the classification performances of training data were computed using 10-fold inner cross-validation. The $C_{v}$ and the new dimension was determined from the value of the cumulative variance at which the best performance was obtained. Using this new dimension, the classification model was built from training data (9 folds), and the test performance was determined from the remaining one fold. This procedure was repeated in each run of 10 fold cross-validation, and average test performance was computed. Table 4 shows the classification performance of different features using SVM and ANN classifiers for each of the six conditions and also for ACT. We observed that the classification performance of all the features was substantially higher than the empirical chance level. Of note, while the theoretical chance level accuracy is 50% as there are only two classes, the chance level accuracy is liable to increase in the presence of a small number of data samples and is referred to as the empirical chance level. It was determined using the method in Combrisson and Jerbi (2015), which assumes that the classification errors obey a cumulative binomial distribution.

Among the wavelet features, the local features performed better than global and hemispheric features; the former yielded a better balance between sensitivity and specificity for both SVM and ANN classifiers. The sensitivity for global and hemisphere-specific features for all condition was higher than the indicating that the transition state was classified more accurately than the maintenance state at the global and hemispheric brain level. The left hemispheric features performed relatively better as compared to that of right hemispheric features indicating a greater contribution of left hemispheric features towards distinguishing the two classes. Alternatively, these two types of features may also capture complementary information. Thus, we performed an analysis by predicting the states by combining the scores obtained from both the features. The integration of the scores from left and right hemispheric features ($S_{l}$ and $S_{r}$) was performed by the given equation,

4 $$\begin{aligned} S_{f} = p*S_{l} + (1-p)*S_{r} \end{aligned}$$

**Fig. 5:**
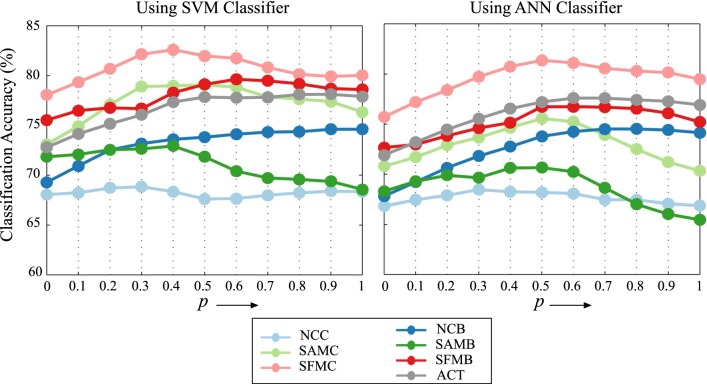
Classification accuracy with SVM and ANN classifier when scores from left and right hemispheric features were combined by weighted average

The parameter *p* was varied from 0 to 1, with a step size of 0.1. Lower the value of *p*, lesser the weight assigned to the left hemispheric features. As *p* was varied from 0 to 1, the classification performance (Fig. 5) attained maximum value for mid-range values of *p*, essentially indicating the contribution of both the features in classification.

**Fig. 6:**
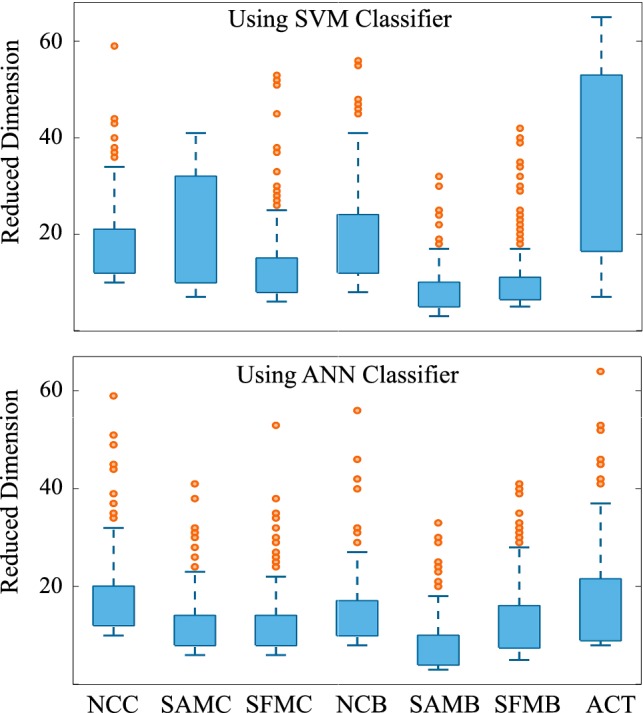
The range of the number of retained principal components for different conditions and for both SVM and ANN classifier is shown by the boxplot

The bias of global and hemispheric features towards classifying the transition state was balanced with the use of local features. We observed an average increase of approximately 9% in specificity as compared to that of the global and hemispheric features. The strength of this approach was its ability to detect both the transition and maintenance state with comparable accuracies. In the case of ANN classifier, a marginal relative improvement in performance as compared to other features was observed. However, the specificity for ACT condition increased by 4.66% and 3.18% using SVM and ANN classifier, respectively. Further, it was found that the reduced dimension of the feature vectors appeared in the range of 3–65 in case of SVM classifier and 3–37 in case of ANN classifier (Fig. 6). It was noted that the number of reduced feature dimension was lesser in each condition as compared to that of ACT condition. This indicated the existence of the variation among the different conditions, which was captured by a comparatively larger number of principle components in ACT.

**Table 4:** Classification performance using different features

Condition	Classification performance (%)							
	Using SVM classifier				Using ANN classifier			
	Accuracy (± SD)	Accuracy above chance-level	Sensitivity	Specificity	Accuracy (± SD)	Accuracy above chance-level	Sensitivity	Specificity
*Global features*								
NCC	68.64 (± 08.11)	14.10	83.18	54.10	68.04 (± 08.72)	13.50	71.21	64.86
SAMC	77.08 (± 12.78)	19.05	75.13	79.02	73.86 (± 14.26)	15.83	76.43	71.28
SFMC	80.28 (± 07.88)	25.40	86.27	74.29	82.57 (± 08.10)	27.69	81.68	83.47
NCB	74.23 (± 07.70)	19.49	81.82	66.64	72.56 (± 08.60)	17.82	74.43	70.69
SAMB	69.65 (± 13.78)	11.81	83.43	55.87	69.18 (± 16.00)	11.34	69.43	68.93
SFMB	77.49 (± 10.42)	20.57	96.60	58.39	76.16 (± 12.81)	19.24	80.43	71.89
ACT	77.53 (± 03.92)	25.23	83.48	71.59	76.91 (± 04.34)	24.61	79.60	74.21
*Left-hemisphere features*								
NCC	67.79 (± 08.99)	13.25	78.48	57.10	67.42 (± 09.07)	12.88	70.80	64.05
SAMC	75.93 (± 14.91)	17.90	79.55	72.30	72.03 (± 15.10)	14.00	73.03	71.03
SFMC	80.68 (± 08.28)	25.80	86.25	75.10	81.30 (± 08.58)	26.42	81.96	80.64
NCB	74.67 (± 08.07)	19.93	84.02	65.32	74.06 (± 08.04)	19.32	74.98	73.13
SAMB	68.38 (± 15.06)	10.54	79.80	56.95	64.80 (± 17.23)	06.96	64.67	64.93
SFMB	78.47 (± 10.58)	21.55	96.70	60.24	75.75 (± 13.04)	18.83	81.73	69.77
ACT	77.74 (± 04.09)	25.44	83.49	71.98	77.30 (± 04.06)	25.00	80.03	74.57
*Right-hemisphere features*								
NCC	67.56 (± 07.70)	13.02	82.28	52.84	67.06 (± 09.22)	12.52	70.96	63.16
SAMC	71.66 (± 16.04)	13.63	61.28	82.03	71.83 (± 15.05)	13.80	72.12	71.55
SFMC	77.91 (± 07.79)	23.03	84.32	71.50	76.81 (± 08.61)	21.93	77.21	76.41
NCB	68.70 (± 09.05)	13.96	80.21	57.20	67.86 (± 08.30)	13.12	70.18	65.54
SAMB	68.21 (± 13.92)	10.37	85.75	50.67	67.66 (± 15.02)	09.82	70.00	65.32
SFMB	77.54 (± 10.65)	20.62	93.40	61.67	72.95 (± 13.85)	16.03	76.99	68.90
ACT	72.18 (± 04.10)	19.88	78.53	65.83	72.32 (± 04.18)	20.02	75.21	69.42
*Local features*								
NCC	76.04 (± 07.80)	21.50	78.27	73.81	73.86 (± 08.89)	19.32	75.56	72.16
SAMC	76.89 (± 13.63)	18.86	75.13	78.65	72.24 (± 13.79)	14.21	71.10	73.38
SFMC	80.85 (± 07.61)	25.97	81.64	80.06	80.93 (± 08.38)	26.05	81.21	80.65
NCB	77.43 (± 07.45)	22.69	81.52	73.33	76.31 (± 07.82)	21.57	76.58	76.04
SAMB	71.21 (± 14.10)	13.37	69.93	72.48	66.43 (± 14.41)	08.59	66.50	66.37
SFMB	79.83 (± 11.80)	22.91	88.23	71.43	75.70 (± 12.04)	18.78	79.26	72.14
ACT	79.58 (± 03.87)	27.28	82.92	76.24	78.40 (± 04.13)	26.10	79.41	77.39
*Local PSD features*								
NCC	50.52 (± 04.17)	− 04.02	94.14	06.90	57.19 (± 08.80)	02.65	60.91	53.48
SAMC	52.63 (± 11.18)	− 05.40	17.03	88.22	52.90 (± 13.73)	− 05.13	47.43	58.37
SFMC	54.56 (± 05.40)	− 00.32	94.84	14.27	58.17 (± 11.46)	03.29	60.49	55.86
NCB	50.23 (± 05.60)	− 04.51	61.00	39.45	53.11 (± 09.48)	− 01.63	51.21	55.02
SAMB	51.31 (± 07.42)	− 06.53	94.63	07.98	52.66 (± 11.86)	− 05.18	53.70	51.62
SFMB	52.06 (± 06.59)	− 04.86	96.29	07.83	52.88 (± 14.52)	− 04.04	58.23	47.52
ACT	51.95 (± 02.14)	− 00.35	96.88	07.02	58.63 (± 05.31)	06.33	60.51	56.75

The classification performance of the wavelet-based features was compared with the performance of the power spectral density features. The power spectral density was estimated in six frequency bands using the Welch’s method. The features were extracted in ten cortical regions and from four frequency band similar to wavelet-based local features. The performance of the features was evaluated using both SVM and ANN classifier, and the results are reported in Table 4. It can be observed that the wavelet-based feature outperformed the power spectral density features, indicating the strength of temporal information captured by the wavelet-based features in discriminating the hidden mental states. The standard deviations of classification accuracies for wavelet as well as power spectral density features were comparatively high for some of the conditions due to the smaller number of samples. However, the ACT condition, which consists of a larger number of samples, yielded a much less standard deviation of accuracy.

### Temporal evolution of internal mental states

**Fig. 7:**
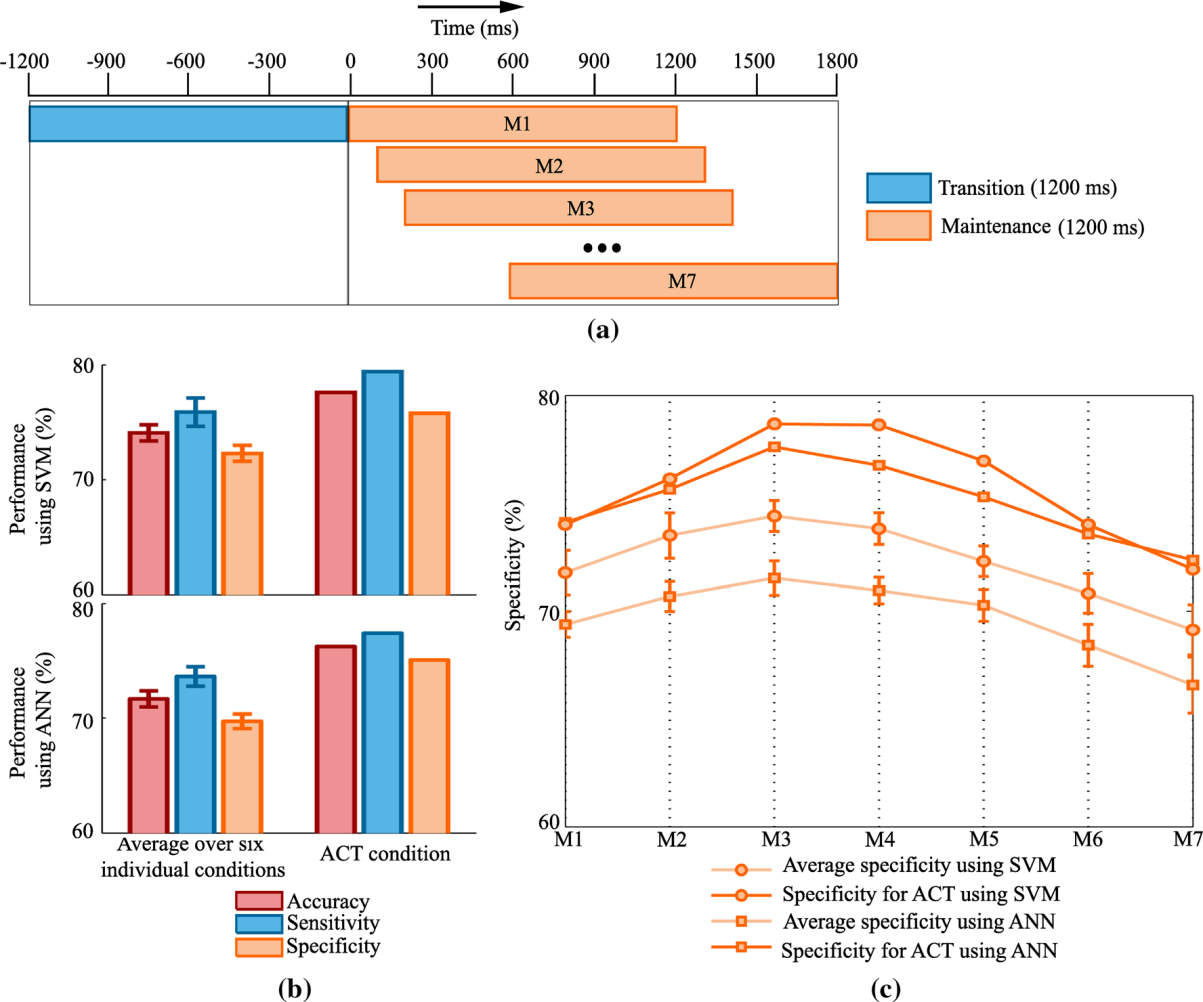
**a** Framework for the temporal evolution of internal mental states. The transition state is considered to be 1200 ms just before the indication of the perceptual switch. Maintenance state is considered to be of the same length as transition state but shifted towards the next transition state seven times with steps of 100 ms. **b** Classification performances quantified by accuracy, sensitivity, and specificity are reported in two ways: classification results averaged over the different conditions and classification performance on the data (ACT) pooled from all different conditions. The error bars indicate the standard error of the mean (SEM) of classification performances for different conditions. **c** Profiles indicate how accurately the maintenance state is detected. The x-axis indicates the different maintenance state, as shown in Fig. 7a

Since the process of transition is expected to occur rapidly, the duration of the transition state is supposed to be short-lived. However, the maintenance state could extend up to the start of the next transition state, and it can be much longer than the considered 1200 ms duration. It would, therefore, be interesting to investigate how precisely we could detect the maintenance state over this extended duration. We explored this possibility by shifting a 1200 ms sub window representation of maintenance state away from the button press with the step size of 100 ms to cover a zone from button press to 1800 ms (Fig. 7a). All these maintenance periods were pooled together to evaluate the performance of the classifier, and the detection performance for each of them was noted. Consequently, the number of trials for the maintenance state was seven times more than that of the transition state. The performance of the classifier was evaluated using local features in the classification framework as described in the previous section in conjunction with random subsampling (Kubat et al. 1997) approach to address the class data imbalance. The classification performance for ACT condition and average performance over all the individual conditions are reported in Fig. 7b. The average accuracy over the individual conditions and accuracy for ACT condition were respectively 74.10% and 77.59% using SVM classifier, 71.66% and 76.23% using ANN classifier. A balance between the sensitivity and specificity was observed in each case. Further, to investigate the effect of different sub-windows of maintenance state on classification performance, the average specificity across conditions and specificity for ACT were plotted against individual subwindows of the maintenance state (Fig. 7c). It was found that the specificity of the classifier first increased up to second sub window, maintained a good classification performance during second to fifth sub window and, decreased afterwards as maintenance state was shifted away from the moment of the perceptual switch. However, the average specificity across the condition and specificity of ACT did not decline below 69.14% and 71.94%, respectively using SVM classifier and were found to be 66.57% and 72.37%, respectively using ANN classifier.

### Role of underlying brain sources in classification of mental states

In this section, we investigate the brain regions involved in the transition and maintenance state. For that purpose, we estimated the time series data in source domain reconstructed from MEG data recorded at sensor level and extracted the features in source domain by following the same feature extraction method as discussed in “Wavelet based features extraction” section and “Source reconstruction” section, respectively. This analysis in source domain would identify the neural sources in the brain rather than its projected effect on the scalp level. We have employed the framework used in the previous section (“Temporal evolution of internal mental states” section) that took into consideration the wider maintenance state duration. We considered local features extracted from reconstructed sources in six frequency bands and 89 brain regions for classification using SVM and ANN classifier. Here, we used *F*-ratio based feature selection technique instead of PCA, as in the latter, data was projected on the direction of principal components that leads to loss of information as regards to one specific source of the feature. *F*-ratio is computed as the ratio of between-class variance to the total within-class variance. A larger *F*-ratio indicates a greater separability between the classes, thus essentially implying more effective feature to discriminate the classes. The features with largest *F*-ratio were given highest priority. Thus, besides the classification performance, this analysis also found discriminative and dominant features by analyzing the features which were selected at the training stage. This, in turn, led to the identification of the underlying neural sources which responded differently during two mental states within a particular frequency band.

**Fig. 8:**
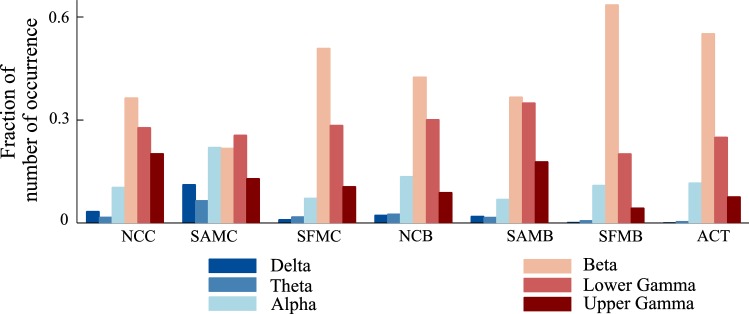
The distribution of the selected features with SVM classifier over six frequency bands

**Fig. 9:**
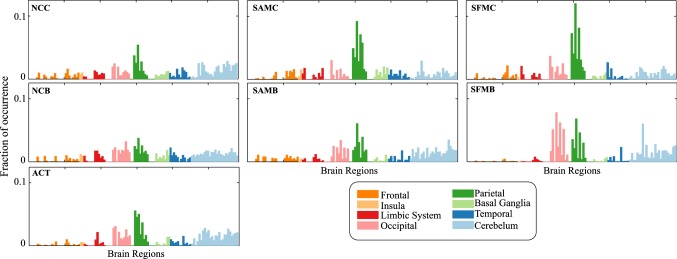
The distribution of the selected features with SVM classifier over different brain regions

**Fig. 10:**
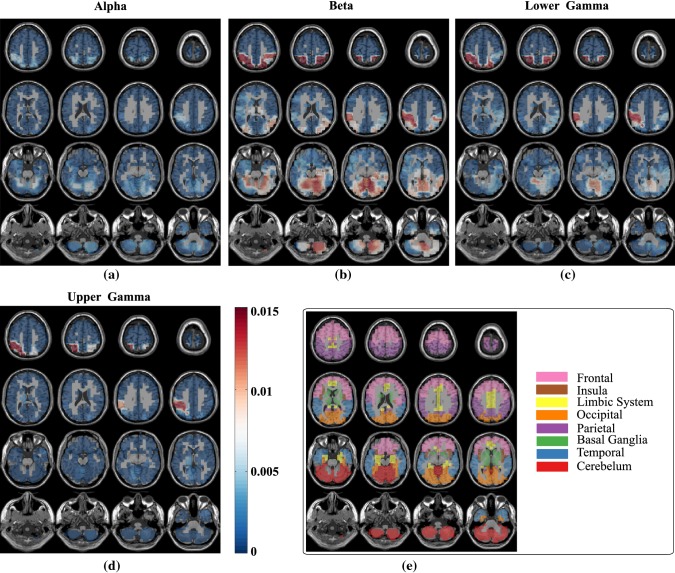
The distribution of the selected features with SVM classifier in **a** alpha, **b** beta, **c** lower gamma and **d** upper gamma bands over the grey matter for ACT condition. **e** Coarse anatomical labeling of grey matter is shown

In this method, we were able to classify the mental states with an average (averaged over six conditions) accuracy of 73.55% and 71.19% using SVM and ANN classifier, respectively; 74.98% and 73.05% accuracy using SVM and ANN classifier, respectively when ACT condition was considered. We analyzed the source features that were selected at the training stage in each fold. Figure 8 shows the distribution of selected features over different frequency bands. The dominance of features from alpha, beta, lower gamma, and upper gamma frequency band was noticed, consolidating our earlier sensor space based findings. The distribution of the selected features over brain regions (Fig. 9) showed that the features were selected in the majority from parietal, occipital and cerebellum area. Thus, the sources of these areas were modulated differently during the state of transition and maintenance. Figure 10 shows the distribution of the selected features over anatomical brain regions for each of the highly contributing frequency bands for ACT conditions. The weights indicate the fraction of the number of selected features from that region. The weights of the excluded regions were set to zero for visualization. The rightmost panel shows the coarse labelling of grey matter according to AAL for reference. The features from parietal regions were found to be consistently selected in the majority from alpha, beta, lower and upper gamma frequency band. Besides this, the occipital and cerebellum regions were found to be modulated distinctly for two considered mental states in alpha, beta and lower gamma frequency bands. The analysis of selected features using SVM and ANN classifier showed a similar result. In Figs. 8, 9 and 10 we have presented the result of the analysis using SVM classifier which are similar for ANN classifier.

### Cross-conditional classification

In the above sections, we evaluated the performance of different features under six conditions (three types of stimuli and two ways of presentation). For evaluating the performance for a condition, the data of that condition was divided into training and testing sets; training data was used to build the model and testing data to evaluate the model performance. Again, in the case of ACT, where the data from all the condition were pooled together, the data were treated irrespective of the corresponding condition and followed the same procedure to evaluate the performance. It is interesting to investigate the generalisation of classifier across different stimulus types. Therefore, in this section, we have considered the cross-conditional classification by building the model using the data of one stimulus and testing on the data of others. Instead of considering all the conditions separately, only three stimuli factors (Necker Cube, Stroboscopic Alternative Motion and Structure From Motion) were taken into account irrespective of the stimuli presentation style. This study reveals the cross-conditional generalization power of the features (Peelen et al. 2010) we used for classification purpose. Table 5 represents the cross-classification performances of sensor-based global, hemispheric and local features using SVM and ANN classifiers. This analysis was extended to senor and source based local features in the framework explained in “Temporal evolution of internal mental states” section and the result is presented in Table 6. It was found that cross-conditional classification performances were comparable with the classification result obtained in the previous three sections. We may conclude that brain responses captured by wavelet features are generalized in discriminating maintenance and transition states across different stimulus.

**Table 5:** Cross-conditional classification performance using different sensor domain features

Features	Classifier	Classification performance (%)								
		Necker cube (NC)			Stroboscopic alternative motion (SAM)			Structure from motion (SFM)		
		Accuracy	Sensitivity	Specificity	Accuracy	Sensitivity	Specificity	Accuracy	Sensitivity	Specificity
Global	SVM	Training			78.97	84.11	73.83	82.07	87.88	76.26
		71.05	82.04	60.06	Training			79.80	86.87	72.73
		70.28	77.40	63.16	74.77	80.37	69.16	Training		
	ANN	Training			78.32	77.48	79.16	81.30	82.47	80.13
		69.88	73.31	66.44	Training			80.09	82.22	77.95
		71.38	73.51	69.24	77.34	78.64	76.03	Training		
Left hemispheric	SVM	Training			78.50	82.24	74.77	81.82	88.38	75.25
		71.05	81.11	60.99	Training			82.83	91.41	74.24
		71.21	78.95	63.47	74.30	77.57	71.03	Training		
	ANN	Training			75.68	77.99	73.36	81.30	84.42	78.18
		71.69	74.72	68.65	Training			81.11	83.31	78.91
		70.65	74.57	66.73	74.39	75.98	72.80	Training		
Right hemispheric	SVM	Training			71.96	78.50	65.42	75.25	84.34	66.16
		64.55	72.45	56.66	Training			74.24	80.81	67.68
		69.81	79.26	60.37	73.36	77.57	69.16	Training		
	ANN	Training			71.45	73.46	69.44	75.96	81.06	70.86
		64.24	67.72	60.76	Training			73.59	76.31	70.86
		67.38	69.41	65.34	72.64	72.85	72.43	Training		
Local	SVM	Training			78.01	80.61	75.42	80.27	84.67	75.86
		67.86	66.30	69.43	Training			77.32	75.53	79.12
		71.54	73.00	70.08	74.91	73.79	76.03	Training		
	ANN	Training			77.06	76.96	77.15	78.12	80.28	75.96
		69.08	71.07	67.09	Training			74.76	75.03	74.49
		72.43	74.04	70.82	76.05	77.06	75.05	Training

**Table 6:** Performance of cross-conditional classification of local features in sensor and source space using the framework explained in “Temporal evolution of internal mental states” section

Classifier	Feature	Classification performance (%)								
		Necker cube (NC)			Stroboscopic alternative motion (SAM)			Structure from motion (SFM)		
		Accuracy	Sensitivity	Specificity	Accuracy	Sensitivity	Specificity	Accuracy	Sensitivity	Specificity
SVM	Sensor	Training			75.32	75.43	75.30	76.24	83.77	74.67
	Source	Training			70.44	76.10	69.64	72.24	85.71	69.42
	Sensor	67.94	66.03	68.21	Training			68.87	74.10	68.45
	Source	67.87	63.51	68.49	Training			69.22	75.54	68.71
	Sensor	72.44	72.05	72.50	72.69	73.03	72.67	Training		
	Source	70.78	63.64	71.80	71.69	71.96	71.68	Training		
ANN	Sensor	Training			75.02	73.56	75.22	75.57	80.74	74.49
	Source	Training			70.84	72.50	70.61	71.70	79.08	70.16
	Sensor	70.07	71.65	69.85	Training			70.67	79.15	69.98
	Source	62.28	65.06	61.88	Training			63.03	73.67	62.17
	Sensor	72.95	71.52	73.15	73.13	71.70	73.19	Training		
	Source	64.80	66.48	64.56	65.03	74.10	64.62	Training		

## Discussion

The primary aim of this study was to decode internal mental states. For this purpose, we have considered bistable stimuli as it involves two internal mental states,  transition and maintenance. The transition state is short-lived and represents the change in perception and maintenance state is of comparatively longer duration and represents one of the two stable perceptions. These two states represent two different internal processes, which we wanted to characterize rather than two stable perceptions which represent two different perceptual states but similar internal processes. Most published studies have focused on decoding external task related demand(s), yet internal mental states are likely to be task independent, and therefore, could fluctuate in an unpredictable fashion. Therefore, decoding internal mental states remain a big challenge.

In this study, we classified the transition and maintenance states involved in bistable perception using MEG data on a single-trial basis. This was done by applying a machine learning framework to the spatiotemporal dynamics of large-scale brain oscillations. To capture this temporal information, three different features were derived (global, hemispheric and local) using wavelet subband energies in the alpha, beta, lower gamma, and upper gamma frequency bands. We also determined the effective transition state by evaluating the classification performance on different lengths of transition and maintenance state. The best classification result was obtained when local region based features were used along with the PCA to reduce feature dimension as well as redundancy. We also investigated the influence of the shift in maintenance state on classification performance. Further, we identified the underlying sources that were regulated differently during transition and maintenance states.

Three types of features were used to classify the maintenance and transition state, namely global, hemispheric and local features. The best classification performance (66.43% to 80.93% for different paradigms using SVM and ANN classifiers) was obtained using local features along with PCA. Using this method, we achieved an increase in specificity, in comparison with the result obtained from global features by using an SVM classifier. We found that when all the trials were pooled together irrespective of conditions for evaluation, good classification performance was obtained for all the feature types. It essentially indicates that the temporal patterns that discriminate the two mental states have similar characteristics across different stimuli and presentation mode. Again, the cross-conditional classification analysis showed the generalization capability of wavelet features of both sensor and source domain to distinguish between transition and maintenance class.

It is noted that the maintenance state persists from the set of previous transition state up to the onset of the next transition state. Hence, the duration of the maintenance state is much longer than that of the transition state. Taking this into consideration, we also demonstrated the temporal evolution of classification performance as maintenance state progressed. It was observed that as the maintenance state was shifted towards the next transition state, the performance accuracy generally decreased. This is because the shifting of maintenance state away from perceptual reversal made it closer to the next transition state. Additionally, it has been reported that resolving uncertainties creates a pleasant jolt in our brain (Ramachandran and Rogers-Ramachandran 2007), the associated activity is captured in the first three subwindows of maintenance state just after the transition state, thus leading to higher performance than the other subwindows.

The findings of our study are largely consistent with earlier studies which show the involvement of parietal areas (Knapen et al. 2011; Megumi et al. 2015), occipital areas (Sterzer et al. 2002) and cerebellum (Calhoun et al. 2001) in bistable perception and visual perception. We found features extracted from these regions were useful to discriminate between the transition and maintenance state. The discriminative information was captured by the spatio-temporal features in alpha, beta, lower gamma, and upper gamma band features.

We focused our analysis on the spectral content of the large-scale brain responses, i.e. on the local synchronization properties of the underlying brain region(s), yet there is evidence of inter-region synchronization during bistable perception (Doesburg et al. 2009; Helfrich et al. 2014). So, future studies could include connectivity measures as suitable features for classification. Here, we considered three different types of ambiguous visual stimuli with each presented in two fashions, continuous and intermittent. Inevitably, there would be differences between these conditions. Yet, the study demonstrates that the underlying internal mental states around the moment of the perceptual switch are possibly similar, and therefore, our classifier was able to achieve a consistent accuracy across these conditions.

In this study, we evaluated the transition and maintenance state classification performance in both sensor and source space. We removed the channels over the motor areas in sensor space. And in source space, we removed the sources that are known for motor-related activity (namely, Precentral Gyrus Left/Right, Superior Frontal Gyrus Left/Right, Middle Frontal Gyrus Left/Right, Inferior Fronto-Opercular Gyrus Left/Right, Rolandic Operculum Left/Right, Supplementary Motor Area Left/Right, Middle Cingulum Gyrus Left/Right, Posterior Cingulum Gyrus Left/Right, Calcarine (Visual) Cortex Left/Right, Cuneus Left/Right, Superior Parietal Gyrus Left, Post-Central Gyrus Left/Right, Pre-Cuneus Left/Right, Para-Central Lobule Left/Right) . One of the limitations of this study is that removing the channels or sources might not entirely eliminate motor-related activity. However, it should reduce motor activity to a large extent. Further, we observe that the discriminating features involved in transition and maintenance mental state classification were mainly from parietal, occipital and cerebellum region. These altogether suggest that our approach of classification is not likely to be due to motor-related activity. However, we acknowledge that future experiment using various types of spontaneously generated actions could be performed for distinguishing these two internal states, maintenance and transition, as studied for bistable perception in the current article from other endogenous processes requiring self-initiated actions, in general.

## Conclusion

Classification of internal mental states from brain signals has become an important challenge in cognitive neuroscience. Here, we present a novel approach to classify two internal mental states during bistable visual perception surrounding the perceptual switch—the transition and maintenance states. We demonstrated that it was possible to classify these states, with an accuracy significantly higher than the chance level. The classification performance was found to be robust against various types of bistable stimuli, thus potentially capturing a general pattern of the perceptual switch. Further, we observed the involvement of sources from the parietal lobe, occipital lobe, and cerebellum in distinguishing between transition and maintenance states. Altogether our results provide a systematic link between brain activity patterns and spontaneously generated internal mental states.

## Footnotes

1 http://www.fmritools.com/kdb/morphological-and-connectom/.
